# Hg tolerance and biouptake of an isolated pigmentation yeast *Rhodotorula mucilaginosa*

**DOI:** 10.1371/journal.pone.0172984

**Published:** 2017-03-02

**Authors:** Bing Liu, Chaogang Wang, Danxia Liu, Ning He, Xu Deng

**Affiliations:** 1 College of Life Sciences and Oceanography, Shenzhen Key Laboratory of Marine Bioresources and Ecology, Shenzhen University, Shenzhen, China; 2 Department of Chemical/Biochemical Engineering, Xiamen University, Xiamen, China; Chinese Academy of Sciences, CHINA

## Abstract

A pigmented yeast R1 with strong tolerance to Hg^2+^ was isolated. Phylogenetic identification based on the analysis of 26S rDNA and ITS revealed R1 is a *Rhodotorula mucilaginosa* species. R1 was able to grow in the presence of 80 mg/L Hg^2+^, but the lag phase was much prolonged compared to its growth in the absence of Hg^2+^. The maximum Hg^2+^ binding capacity of R1 was 69.9 mg/g, and dead cells could bind 15% more Hg^2+^ than living cells. Presence of organic substances drastically reduced bioavailability of Hg^2+^ and subsequently decreased Hg^2+^ removal ratio from aqueous solution, but this adverse effect could be remarkably alleviated by the simultaneous process of cell propagation and Hg^2+^ biouptake with actively growing R1. Furthermore, among the functional groups involved in Hg^2+^ binding, carboxyl group contributed the most, followed by amino & hydroxyl group and phosphate group. XPS analysis disclosed the mercury species bound on yeast cells was HgCl_2_ rather than HgO or Hg^0^.

## Introduction

Heavy metal pollutants are generated through a wide range of industrial activities and continue to be released into the environment at harmful quantities[1]. Pitfalls of non-biological approaches to heavy metal removal make a microbial-based technology for the detoxification of heavy metal in polluted systems a cost-effective and more environmentally friendly remediation option[2]. Although typically bacteria are commonly used in bioremediation studies, there are more fungal studies now than ever[3]. As with bacteria, fungi can naturally develop modified metabolism to deal with environmental contaminants and then be used in bioremediation[4]. Yeasts are good examples of fungi having a body size larger than bacteria. Like other eukaryotic organisms, yeasts have a nucleus and associated cytoplasmic organelles. The cytoplasm in living cells is responsible for the interactions with metal ions. Although yeast cells are generally known to be mediocre in terms of metal biouptake[5], recently the use of yeasts cells for accumulating heavy metals has gained more ground[6–8].

*Rhodotorula* species, which belong to the phylum *Basidiomycota*, are found to be common in natural environment and have been isolated from industrial and municipal wastes as well as polluted areas. A number of *Rhodotorula* species have been confirmed to be able to remediate some specific contaminants. For example, *R*.*glutinis* and *R*.*rubra* have both been found to have a high ability to degrade phenanthrene, while *R*.*minuta* was able to degrade benzo(a)anthracene. In a mixed fungal community *Rhodotorula* species contributed to effective degradation of low molecular weight PAHs, and although bacterial communities alone were not able to, the fungal communities also degraded high molecular weight PAHs (more than 3 benzene rings) such as chrysene and benzo(a)pyrene [9–11]. These results exhibit the promising potential of *Rhodotorula* species in the field of bioremediation[11]. For heavy metal bioremediation, despite previous studies on Cu tolerance and detoxification by *Rhodotorula* sp[12–13], few investigations on Hg tolerance and biouptake of these species were performed to our knowledge. Therefore, the biouptake of Hg could be a new use for this species.

In the present study, *Rhodotorula mucilaginosa* R1, a Hg-tolerant yeast was isolated and identified. The analysis of its tolerance to Hg and uptake of this metal was performed. Furthermore, the effect of coexisting organic substances on Hg uptake of R1 was evaluated, and the strategy to remove Hg from multi-component aqueous environment was developed. Finally, the mechanism of Hg biouptake by the yeast cells was explored based on chemical modification of functional groups and XPS analysis.

## Materials and methods

### Yeast isolation

Sediment samples were separately collected from Shenzhen Xixiang river, China. No specific permissions are required for these activities because it is a public area andthe field studies do not involve endangered or protected species. The samples were serially diluted and plated onto solid LB culturing medium (peptone, 10 g/L; yeast extract, 5 g/L; NaCl, 10 g/L; agar, 15 g/L) amended with 20 mg/L Hg^2+^ in the form of HgCl_2_ and incubated at 30°C for 3 d to allow the appearance of red colonies. The red colonies were further purified by repeated streaking on YPD (yeast extract 10 g/L, peptone 20 g/L, dextrose 20 g/L, pH 7.5) agar plates supplemented with 20–100 mg/L Hg^2+^ and incubated at 30°C for 2–7 d.

### Phylogenetic identification of the isolate

One mercury-tolerant yeast isolate was selected for further studies and its phylogenetic identity was determined by analysis of rRNA gene sequences. Total genomic DNA from the strain was prepared according to the method used by Tristezza et al.[14]. The D1 and D2 regions of 26S ribosomal DNA (rDNA) and internal transcribed spacer (ITS) regions in the rRNA gene were sequenced directly from PCR products using the primer pairs NL-1 (forward; GCATATCAATAAGCGGAGGAAAAG) and NL-4 (reverse; GGTCCGTGTTTCAAGACGG) and ITS1 (forward, TCCGTAGGTGAACCTGCGG) and ITS4 (reverse, TCCTCCGCTTATTGATATGC), respectively.PCR was performed at 94°C for 30 s; 55°C for 30 s; and 72°C for 1.5 min, for 30 cycles, and then followed by a final extension at 72°C for 10min. After agarose gel analysis, the amplicons were purified and then sequenced. The search for sequence similarity with sequences in the GenBank database was performed by using the BLASTN algorithm. Strains with 99% or more similarity of the D1 and D2 regions of 26S rDNA and the overall ITS sequences were defined as conspecific [15]. A phylogenetic tree based on 26S rDNA was constructed using the neighbour-joining method. Furthermore, bootstrap analysis was performed to assess the confidence limits of the branching. The nucleotide sequences of 26S rDNA and ITS determined in this study have been deposited in GenBank under the accession number KU094060 and KU094061, respectively.

### Mercury tolerance of the isolated yeast

Tolerance to mercury was tested by growth of yeast cells in 0.1YPD (yeast extract 1 g/L, peptone 2 g/L, dextrose 2 g/L, pH7.5) liquid medium supplemented with increasing concentrations of Hg^2+^ from 20 mg/L to 100 mg/L at 30°C, 150rpm for 2–7 d. In our experiment, 0.1YPD medium was used in an attempt to replicate relatively poor nutrition condition commonly existing in heavy metal wastewaters. A brewing *Saccharomyces cerevisiae* strain, deposited in our lab, was used as the control. Growth was monitored by optical density measurements at 600 nm. To determine the dry weight of the biomass, 20 mg sample of wet biomass was dried at 110°C to a constant weight.

### Mercury biouptake

To obtain biouptake isotherm of the strains, yeast cells were cultured overnight at 30°C, 150 rpm in YPD medium. Then the cells were harvested by centrifugation at 8000×g for 10 min, washed three times with 0.85% NaCl solution, and resuspended in ddH_2_O solutions with the desired Hg^2+^ concentrations. After 2 h incubation at 30°C, 150 rpm, the cells were removed and pelleted by centrifugation. Then the supernatants and the pelleted cells were measured for the quantity of Hg^2+^. For supernatant samples, 5% HNO_3_-0.05% K_2_Cr_2_O_7_ was added before measurement to minimize Hg^2+^ loss due to glassware adsorption. For cell samples, the cells were pelleted, dried, and digested overnight with 70% trace-metal grade nitric acid for Hg^2+^ analysis.

The uptake of mercury by living and dead yeast cells over a period of 2h was examined by separately suspending equivalent dose of viable and dead cells in 10 mg/L Hg^2+^ solutions. Dead yeast cells were obtained by treating the harvested cells in autoclave at 105°C for 15 min.

To test mercury biouptake behavior in multi-component aqueous solution, equivalent dose of yeast cells were suspended in ddH_2_O (control), 0.1YPD medium and 0.01YPD medium (peptone, 0.2 g/L; yeast extract, 0.1 g/L; dextrose, 0.2 g/L), respectively supplemented with 10 mg/L Hg^2+^. After 2 h incubation at 30°C, 150 rpm, the cells were pelleted. Then the supernatants and pelleted cells were measured for Hg^2+^ as described above.

Mercury uptake by actively growing yeast cells was examined in cultures propagated in 0.1YPD medium with Hg^2+^added to a final concentration of 10 mg/L at 30°C, 150 rpm for 30 h. During cultivation process the samples were taken out at the different time intervals until the cells reach their stable-state phase. The cell density of samples was first measured as OD_600_ by a spectrophotometer at 600 nm. Then the samples were centrifuged, and the resultant cell pellets and supernatants were measured for Hg^2+^ determination as stated above.

All experiments were carried out in triplicate. All glassware was soaked in 20% nitric acid overnight and rinsed three times with ddH_2_O before complete drying.

### Participation of functional groups in Hg^2+^ uptake

To understand the role of functional groups in metal ion binding, biomasses were chemically treated in different ways. Cells were suspended for 30 min in anhydrous methanol and concentrated hydrochloric acid (1:1, v/v) to esterify carboxylic groups, in formaldehyde and formic acid (1:1, v/v) to methylate amine or hydroxyl groups, and in triethyl phosphate and nitromethane (1:1, v/v) to esterify phosphate groups. After chemical modification, the cells were washed twice with ddH_2_O, harvested by centrifugation at 8000×g for 10 min and resuspended in10 mg/L Hg^2+^ solution. The decrease in metal uptake capacity of treated cells was used to evaluate the contribution of blocked functional group in the overall biouptake process [16].

### X-ray Photo-electron Spectroscopy (XPS) analysis

XPS analysis of mercury-laden/unladen cells was performed according to the method described before [17–18]. Yeast cell samples were washed with ddH_2_O, frozen in liquid nitrogen, freeze-drying at 268 K, mounted the obtained powder in a trough and then pressed before being transferred to the analysis chamber of the spectrometer.

### Analytical methods

A sequential inductively coupled plasma optical emission spectrometer (ICP-OES, OPTIMA 7000, Perkin Elmer) was employed to determine Hg^2+^. The dry weight of cells was calculated from the OD_600_ by using the value of 0.292 g dry weight per liter at an OD_600_ of 1.0 which was obtained in the experiment. Hg^2+^ binding capacity was expressed as milligram Hg^2+^ accumulated per gram dry weight of cells (mg/g). Hg^2+^ removal ratio was expressed as the percentage of Hg^2+^ removed from Hg^2+^ aqueous solution (%). XPS analysis was performed by a X-ray photo-electron spectroscopy (Microlab 350, Thermo VG).

### Statistical analysis

Results were expressed as the mean plus or minus the standard deviation. Statistical comparison was performed using one-way analysis of variance. A probability (P) value of less than 0.05 was considered statistically significant.

## Results

### Strain isolation and identification

**Fig 1:**
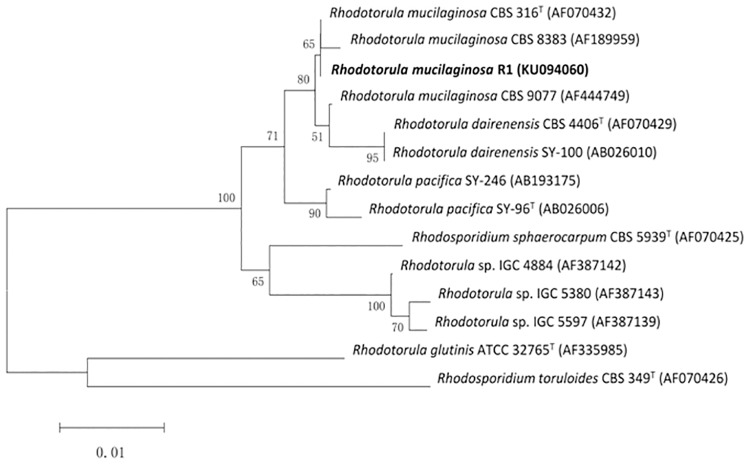
Phylogenetic tree. Bootstrap values (expressed as percentages of 1000 replications) are shown at the branch points. Bar, 0.01 nucleotide substitution rate (Knuc) units.

A yeast isolate obtained from sediment enrichment culture, designated as R1, was selected on the basis of its tolerance to Hg^2+^. The sequence of 26S rRNA gene (D1/D2 region) showed 99% homology with *Rhodotorula mucilaginosa* (accession no: EU7076.1) in similarity search using BLAST program. Furthermore, ITS1 and ITS2 region showed 99% homology with *Rhodotorula mucilaginosa* (accession no: AB026003.1). In the phylogenetic tree of the isolated yeast with other closely related strains established in Fig 1, the isolate is near *Rhodotorula mucilaginosa* CBS8383 (AF189959). Therefore, the isolate R1 is identified as a *Rhodotorula mucilaginosa* strain.

### Tolerance of R1 to mercury

**Fig 2:**
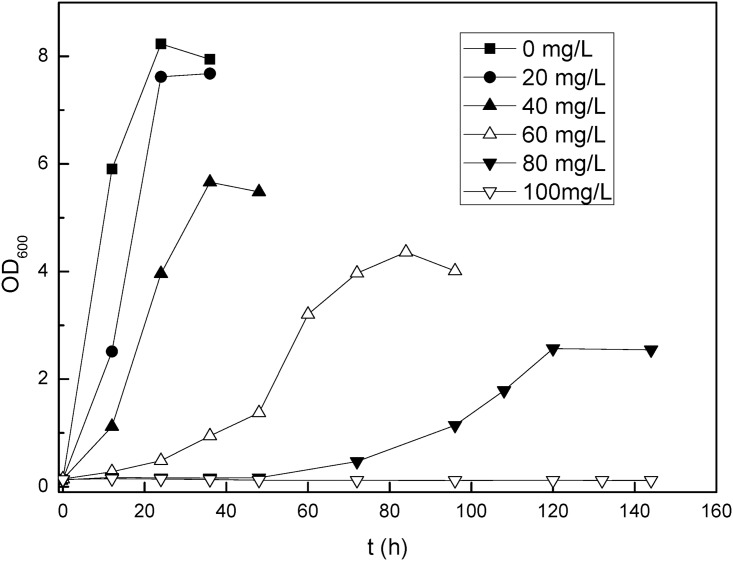
Growth of R1 in 0.1 YPD medium complemented with different concentration of Hg^2+^.

Fig 2 showed the growth of R1 in 0.1YPD medium supplemented with different concentrations of Hg^2+^. It was clear that the presence of 20 mg/L Hg^2+^ didn’t visibly affect R1 growth. However, with the increase of Hg^2+^ concentration, the inhibition of toxic mercury on the growth of R1 showed up by extending the lag phase and decreasing the maximum OD_600_ it could reach. As Hg^2+^ concentration was 80 mg/L, the lag phase was prolonged to 120 h and the maximum OD_600_ dropped to lower than 3.0, almost 60% decrease compared to that at 20 mg/L Hg^2+^. When Hg^2+^ concentration came up to 100 mg/L, no growth of R1 was observed. On the contrary, the brewing *S*.*cerevisiae*, which was used as the control in our experiment, was not able to grow in the presence of 20 mg/L Hg^2+^ (data not shown), possibly suggesting no mercury detoxification strategy existing in brewing *S*.*cerevisiae*.

### Biouptake capacity of Hg^2+^

Our experimental results revealed that Hg^2+^ bioaccumulated by yeast cells at equilibrium (q, mg/g) as a function of relevant residual Hg^2+^ concentration (C_e_, mg/L) can be expressed by the Langmuir model in a linear form to calculate the maximum Hg^2+^ accumulation capacity (q_m_, mg/g). It was found that experimental data were in good agreement with the empirical Langmuir model, and the q_m_ can be calculated to be 69.9 mg/g and 57.5 mg/g for R1 and *S*.*cerevisiae*, respectively (Table 1).

**Table 1:** Equilibrium biouptake of Hg^2+^.

	C_e_(mg/L)	q (mg/g)	Langmuir model	1/q_m_	q_m_(mg/g)
**R1**	9.51	35.90	C_e_/q = 0.0143×C_e_+0.0342	0.0143	69.90
	19.57	55.94			
	39.53	62.34			
	59.67	65.00			
	80.54	68.48			
	101.8	67.83			
***S*.*cerevisiae***	12.29	46.06	C_e_/q = 0.0174×C_e_+0.0286	0.0174	57.50
	22.13	54.79			
	42.46	61.85			
	63.21	58.40			
	84.24	61.97			
	106.1	62.93			

### Biouptake in multi-component aqueous solutions

**Fig 3:**
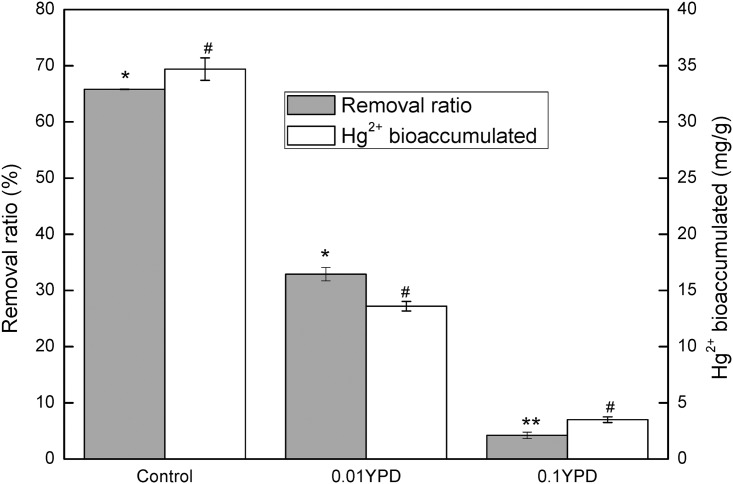
Comparison of Hg^2+^ removal ratio and Hg^2+^ biouptake by R1 from aqueous solutions containing different concentrations of organic substances. The control was made of ddH_2_O and contained no organic substance. 0.1YPD and 0.01YPD represented diluted (1:10 and 1:100) YPD media. All solutions were complemented with 10 mg/L Hg^2+^.

Fig 3 disclosed the remarkable inhibition of organic substances on Hg^2+^ biouptake. Compared with the control, Hg^2+^ removal ratio and Hg^2+^ accumulated by yeast cells decreased 50% and 61%, respectively in 0.01 YPD where the concentration of organic compounds was at a low level. As the concentration of organic compounds increased, only 4.2% Hg^2+^ could be removed from 0.1 YPD medium, almost 94% less than that of the control. Hg^2+^ biouptake capacity of R1 dropped to 3.5 mg/g, accounting for only 10% of that obtained in the control.

**Fig 4:**
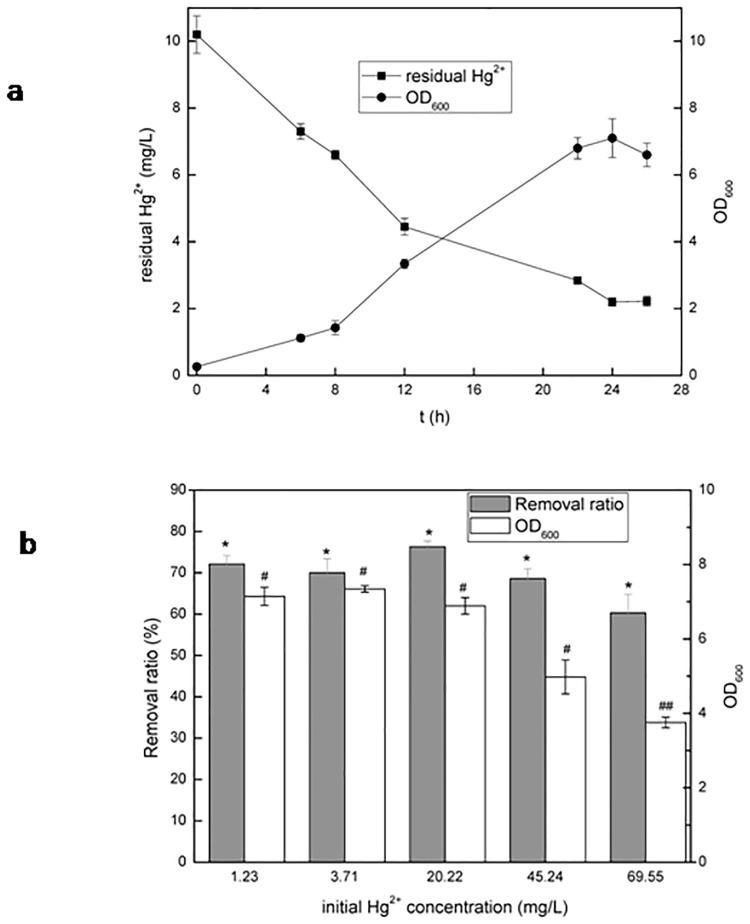
Simultaneous process of cell propagation and Hg^2+^ removal by actively growing R1 from 0.1YPD media. a: Time course of the simultaneous process of cell propagation and Hg^2+^ removal. 0.1YPD medium was complemented with 10 mg/L Hg^2+^. b: Comparison of Hg^2+^ removal and cell growth by actively growing R1 under different concentrations of Hg^2+^ after 30 h cultivation.

The result in Fig 4a showed that R1 cells could grow well in 0.1 YPD containing 10 mg/L Hg^2+^. In 24 h OD_600_ rose up to 6.6. In the meantime, the residual Hg^2+^ concentration of the medium dropped to 2.2 mg/L, demonstrating that 78% Hg^2+^ was removed from the solution, which was much higher than 4.2% obtained by resting cells from 0.1 YPD. Fig 4b exhibited Hg^2+^ removal ratio and OD_600_ that actively growing R1 was able to achieve at different Hg^2+^concentrations. The removal ratio of Hg^2+^ could exceed 60% even when initial Hg^2+^ concentration was 70 mg/L, suggesting that actively growing R1 cells can effectively remove Hg^2+^ from multi-component aqueous solutions.

### Role of functional groups in Hg^2+^ binding

**Fig 5:**
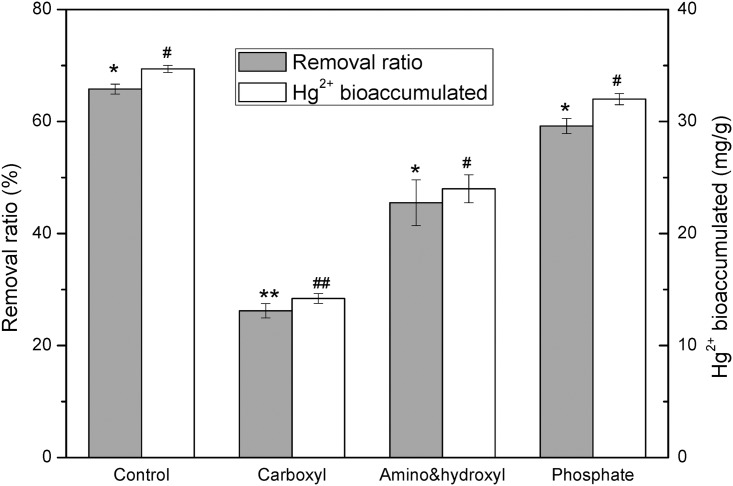
Hg^2+^ removal and Hg^2+^ biouptake from 10 mg /L Hg^2+^ solution by unmodified R1 cells (control) and R1 chemically modified to block carboxyl, amino and hydroxyl, and phosphate groups, respectively. After chemical modification, the cells were washed twice with ddH_2_O, harvested by centrifugation and resuspended in10 mg/L Hg^2+^ solution for 2 h biouptake.

It was clear that carboxyl group was the most important group for Hg^2+^ uptake (Fig 5). The esterification of carboxyl group resulted in decrease of Hg^2+^ removal ratio and Hg^2+^ biouptake capacity from 65.8% and 34.7 mg/g to 26.2% and 14.2 mg/g, respectively in comparison to the control. Blocking of amino & hydroxyl groups did not affect Hg^2+^ uptake of R1 too much since 45.5% of Hg^2+^ removal ratio and 24 mg/g Hg^2+^ uptake remained. On the other hand, phosphate group seemed to slightly contribute to Hg^2+^ binding. Only 10% decrease for both Hg^2+^removal ratio and Hg^2+^ binding capacity occurred after phosphate groups were chemically modified.

### XPS analysis

**Fig 6:**
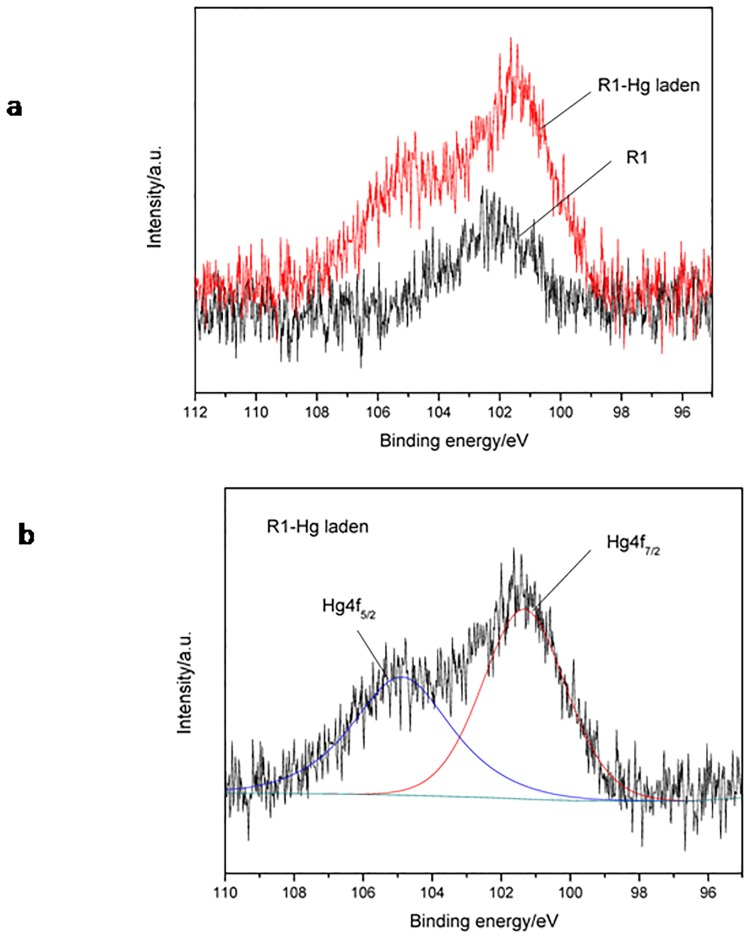
XPS analysis of R1 before/after Hg^2+^ uptake. a: XPS spectra of R1 and Hg-laden R1. b: Calculation of peak area based on origin fitting.

R1 before/after Hg^2+^ uptake was detected by XPS to analyze mercury bound on the cells. Fig 6a showed after Hg^2+^ uptake, Hg^2+^-laden R1 displayed double peaks, whilst original R1 cells had only one peak. In mercury standard spectra, there are supposed to be two peaks in the range of binding energy from 97 to 105 eV, 4f_7/2_ and 4f_5/2_, with the ratio of peak area being 4:3[19]. By fitting the peaks via Origin software and calculating the peak area (Fig 6b), it was found that the area of peak 4f_7/2_ and 4f_5/2_ was 3802.98 and 3005.59, respectively, accounting for the ratio nearly of 4:3. Therefore, this result confirmed mercury binding on R1 cells. Furthermore, mercury species can be figured out based on the binding energy of peak 4f_7/2_. It was reported that the binding energies of Hg 4f_7/2_ of the most appropriate reference compounds are as follows: 101.4 eV(HgCl_2_); 108.0 eV (HgO) and 99.8 (Hg^0^)[17]. Fig 6 disclosed the binding energy of 4f_7/2_ peak was 101.34 eV, clearly shifted beyond the 99.8 eV and 108.0eV reference point for Hg^0^ and HgO, respectively, suggesting mercury species bound on R1 cells might be HgCl_2_ rather than Hg^0^or HgO.

## Discussion

In some previous studies, Cu tolerance and detoxification by *Rhodotorula sp*[12–13] were reported. However, few investigations on Hg tolerance and biouptake of these species were performed. Therefore, a Hg-resistant yeast R1, identified to be *Rhodotorula mucilaginosa* species, was isolated and identified. Our experiment showed that R1 was able to grow in the presence of 80 mg/L Hg^2+^, much higher than 10 mg/L which is the maximum tolerant Hg^2+^ concentration for most reported Hg^2+^-tolerant microorganisms [20], displaying a high tolerance to toxic mercury.

In our experiment, 0.1 YPD medium was used to replicate the relatively poor nutrition condition commonly existing in heavy metal wastewaters. It was reported nutrient condition affects the tolerance of microorganism to heavy metals. Okino et al. [21] found that a *Pseudomonas sp*. was able to grow in LB medium with 100 mg/L Hg^2+^, whereas it could not grow in 0.1 LB supplemented with 40 mg/L Hg^2+^. The tolerance of R1 isolate to Hg^2+^ might be attributed to the presence of heavy metals in Shenzhen Bay [22]. The Hg resistance of some previously reported microorganisms including bacteria and yeasts is listed in Table 2.

**Table 2:** Comparison of mercury resistance of reported microorganisms.

	Strain	MIC * (mg/L)	Reference
**Bacteria**	*Pseudomonas putida*	<10	[20]
	*Pseudomonas sp*.	100	[21]
	*Bacillus thruingiensis PW-5*	50	[23]
	*Bacillus cereus MMRF-575*	100	[24]
	*Bacillus sp*. *AZ-1*	20	[25]
	*Alcaligenes faecalis CH07*	75	[26]
	*Pseudomonas sp*.	120	[27]
	*Halobacterial strain*	25	[28]
**Yeasts**	*Rhodoturula rubra*	6	[29]
	*Yarrowia sp*.	32	[30]
	R1	80	This study

* MIC: Minimum inhibitory concentration of Hg^2+^

Organisms routinely exposed to heavy metal in their ecological niches are subjected to developing tolerance strategies such as bioreduction [23], surface biosorption and biosequestration [24]. Bacterial strains that harbor *mer*A gene are known to reduce Hg^2+^ to volatile Hg0 and thus show the tolerance[25]. For yeast species, Oyetibo et al [30] found two mercury-resistant yeast strains, identified as *Yarrowia* species, were able to reduce and vaporize supplemented Hg^2+^ as metallic mercury (Hg^0^) and consequently showed tolerance to 32 mg L^-1^ Hg^2+^. They postulated Hg^2+^ reduction to Hg^0^ and Hg^0^ volatilization were likely triggered by sulfhydryl compounds present in the biomolecules in response to Hg toxicity. Another mechanism of resistant yeasts responsible for high level tolerance to heavy metal can be the production of some metabolites, such as organic acids, carotenoids and siderophores [13]. On the other hand, yeast cells can sequester heavy metal in vacuoles to prevent toxicity[31].

According to Xu’s investigation, Hg^2+^ concentration of seawater sampled from Shenzhen Bay was in the range of 0.001–0.01 mg/L [22]. Although Hg^2+^ concentrations in contaminated sites are normally not as high as that used in our study, using high Hg^2+^ concentration to screen high Hg^2+^-tolerant microorganisms may facilitate the application of bioremediation of Hg contamination.

The maximum Hg^2+^ binding capacity of R1 is 69.9mg/g, which is not high in comparison to Hg^2+^ binding capacity of other microorganisms [27,30]. Although q_m_ of R1 was about 20% higher than that of *S*.*cerevisiae*, the difference in Hg^2+^ binding capacity between two kinds of yeasts was much smaller than the difference in their Hg^2+^tolerance, suggesting that high tolerance to heavy metal doesn’t necessarily accompany with high binding capacity for microorganisms [32]. For example, bacterial strains with *mer*A gene normally show tolerance to Hg^2+^, but they may not accumulate much Hg^2+^ since Hg^2+^ tends to be reduced to volatile Hg^0^. As a kind of pigmented yeast, *R*. *mucilaginosa* produces carotenoid which has a demonstrated role in protecting the cells from oxidative damage caused by heavy metal [33]. However, Production of carotenoids can be part of a physiological response triggered to avoid cellular accumulation of heavy metals. Irazusta et al.[12] found that there is an inverse relationship between carotenoid production and copper biouptake by a *R*. *mucilaginosa* strain. Furthermore, the adverse effect of such antioxidants on Hg^2+^ accumulation of R1 could partly be confirmed by the difference in Hg^2+^ uptake between dead cells and living cells. Our experiment result showed that dead R1 cells could bind 40.2 ± 0.7 mg g^-1^ Hg^2+^, which is 15% more Hg^2+^ than 34 ± 0.3 mg g^-1^ obtained by living cells from 10 mg L^-1^ Hg^2+^ solution. Only living cells produce carotenoid and subsequently hinder Hg^2+^ binding whereas dead cells will not.

In previous studies, biouptake of heavy metal was mostly evaluated in experimental surrogates that only contained target heavy metal ions. However, actual heavy metal wastewater is much more complicated. There are normally other coexisting metal ions. Their effect on biouptake of target metal has been widely studied [27,34]. On the other hand, organic substances, though at relatively low concentrations, may also be contained but their effect has rarely been focused. Therefore, we evaluated biouptake of Hg^2+^ by R1 in 0.1 YPD and 0.01 YPD media.

The result in Fig 3 showed that the presence of organic substances might remarkably inhibit Hg^2+^ biouptake. YPD medium contains peptone and yeast extract, which are composed of lots of organic compounds such as amino acids, peptides, saccharides, etc. Some of them are able to form chelates or coordination compounds with transition metals, possibly decreasing bioavailability of heavy metals. From the sharp decrease of Hg^2+^ removal ratio caused by the presence of organic matters, it could be deduced that the formed mercury chelates or coordination compounds were soluble but not bioavailable and thus remained in the solution. Obviously this may increase the difficulty in bioremoval of heavy metal from wastewater.

However, the living cells used in Fig 3 is a kind of resting cells (metabolically active but not multiplying) since 2 h of incubation time is not long enough for them to grow and reproduce [30]. Indeed, most of organic compounds are nutrients for microorganisms. If microbial cells possess tolerance to heavy metal, they can make use of organic compounds for growth and propagation and thus increase the bioavailability of heavy metal. It was reported that growing cells may show a greater capacity for the removal of metals than non-viable biomass, especially in environments with nutrients [7]. Actually the results in Fig 4 confirmed that actively growing R1 cells can effectively remove Hg^2+^ from multi-component aqueous solutions.

Binding of heavy metals on different microorganisms may involve different functional groups to varying extents. Since carboxyl group is easier to be deprotonated than other groups [16], it seems to be more competitive in metal binding. Kapoor and Viraraghavan [35] demonstrated that carboxyl and amino groups were important functional groups involved in biosorption of Pb^2+^, Cd^2+^ and Cu^2+^. Carboxyl group was also confirmed to be the most important functional group for a marine *Pseudomonas* sp to uptake Hg^2+^ followed by amino & hydroxyl and phosphate groups to a nearly equivalent extent [27]. However, when using *E*.*coli* JM109 to accumulate Ni^2+^, the contribution of phosphate group was found to be the most important, while carboxyl group contributed the least [36]. For R1 yeast cells, carboxyl group was proved to be the most important functional group. Considering that carboxyl is the easiest deprotonation functional group among the studied groups, it was plausible to postulate that ion-exchange might be the principal mechanism for R1 to accumulate Hg^2+^.

Furthermore, by XPS analysis, mercury binding on R1 cells was confirmed and the species of bound mercury might be mercuric Hg rather than element Hg or HgO. However, since the 4f_7/2_ binding energy for the most common reference materials range only from 99.9 to 101.4 eV and the *Δ*eV (the distance between the 4f_5/2_ and 4f_7/2_ peaks) is consistently 4.1±0.1 eV [37], the confirmation of mercury species bound on R1 cells may need more evidence.

As remediation of heavy metal pollution from local environment can be achieved by using heavy metal-tolerant microorganisms, the yeast R1 cells isolated in this study may have the potential for in-situ bioremediation of mercury contamination.

## Conclusions

A *Rhodotorula mucilaginosa* R1with high tolerance to Hg^2+^ was isolated and identified. R1 could grow in 80 mg/L Hg^2+^, but its Hg^2+^ biouptake capacity was relatively low, suggesting no connection between metal tolerance and accumulation capacity. Organic substances reduced Hg^2+^ bioavailability and thus decreased Hg^2+^ removal, but actively growing R1 cells could consume organic matters and consequently accumulate Hg^2+^ in the solution. Carboxyl group proved to be the most important functional group in Hg^2+^ uptake, while phosphate group contributed the least. XPS analysis confirmed the binding of mercury on R1 and the mercury species being HgCl_2_.
